# Zeolites and Biochar Modulate Olive Fruit and Oil Polyphenolic Profile

**DOI:** 10.3390/antiox11071332

**Published:** 2022-07-06

**Authors:** Sandra Martins, Ermelinda Silva, Cátia Brito, Carlos Martins-Gomes, Alexandre Gonçalves, Margarida Arrobas, Manuel Ângelo Rodrigues, Carlos M. Correia, Fernando M. Nunes

**Affiliations:** 1 CITAB—Centre for the Research and Technology of Agro-Environmental and Biological Sciences, University of Trás-os-Montes e Alto Douro, 5000-801 Vila Real, Portugal; scpmartins@utad.pt (S.M.); emsilva@utad.pt (E.S.); cvqbrito@utad.pt (C.B.); camgomes@utad.pt (C.M.-G.); 2 Association BLC3—Technology and Innovation Campus, Centre Bio R&D Unit, Rua Comendador Emílio Augusto Pires, 14, Edifício SIDE UP, 5340-257 Macedo de Cavaleiros, Portugal; 3 CQ-VR—Food and Wine Chemistry Laboratory, Chemistry Research Centre—Vila Real, University of Trás-os-Montes e Alto Douro, 5000-801 Vila Real, Portugal; 4 MORE—Collaborative Laboratory Mountains of Research, Brigantia Ecopark, 5300-358 Bragança, Portugal; agoncalves@morecolab.pt; 5 CIMO—Centro de Investigação de Montanha, Instituto Politécnico de Bragança, 5300-253 Bragança, Portugal; marrobas@ipb.pt (M.A.); angelor@ipb.pt (M.Â.R.)

**Keywords:** *Olea europaea* L., olive oil quality, rainfed orchards, soil amendments, soil fertility

## Abstract

Soil degradation processes and climate change threaten the sustainability of Mediterranean rainfed olive orchards, with repercussions on crop yield and quality of olives, olive oil and olive by-products. Using soil amendments can enhance soil fertility for sustained environmental quality and plant performance. For two years, we evaluated, under rainfed conditions, the effects of a fertilizer compound (FC) and its combination with zeolites (ZL) and biochar (BC) amendments on soil moisture, yield, fruit and oil polyphenols and quality indices. The polyphenolic composition was strongly influenced by treatments, although no effects were observed on crop yield. ZL improved soil moisture (average increase of 26.3% compared to FC), fruit fatty acid composition (increase of 12.4% in oleic/linoleic ratio in 2018) and oil quality, BC enhanced the concentrations of polyphenols with high nutritional value (average annual increase of 25.6, 84.8 and 11.6% for 3,4-dihydroxyphenylglycol, oleuropein and rutin, respectively). In contrast, olive oil from FC fruits showed the poorest quality, with oxidation and hydrolytic breakdown signals. The applied soil amendments appear to be a promising sustainable strategy to implement in olive rainfed orchards.

## 1. Introduction

The perennial evergreen olive tree (*Olea europaea* L.) is one of the most important crops of the Mediterranean region, where a significant part of the olive oil world’s supply is produced [1,2,3]. Olive oil is widely known as the primary source of fat in the Mediterranean diet, associated with several beneficial effects on human health due to its balanced fatty acid composition and antioxidant properties [4,5]. It is composed mainly of triglycerides [6] and contains small quantities of sterols [7], fatty alcohols [8], carotenes and chlorophylls [9], n-alkanes and n-alkenes [10], phenolic compounds [11] and volatiles [12]. The glyceride fraction presents a high content of fatty acids, particularly an elevated proportion of monounsaturated fatty acids (MUFA) due to its high content of oleic acid (C18:1) and polyunsaturated fatty acids (PUFA), including linoleic (C18:2), linolenic (C18:3) and palmitoleic (C16:1) acids, and low proportion of saturated fatty acids (SFA), including palmitic (C16:0) and stearic (C18:0) acids [5]. The profile and composition of phenolic compounds vary considerably from olive fruit to olive oil due the phenolic oxidation promoted by the activity of various hydrolytic enzymes during the crushing and malaxation processes [13]. Phenolic compounds are indispensable for olive oil quality as they provide remarkable stability against oxidation and are associated with organoleptic and nutritional properties due to their antioxidant, anti-inflammatory and antimicrobial activity [5,14]. For these reasons, the production and consumption of olive oil are increasing worldwide [4].

Despite the high production, the agricultural soils in these areas are typically characterized by low organic matter content and are exposed to severe degradation and persistent loss of fertility [15]. A decline in soil quality has a marked impact on tree growth, yield, olive oil quality and production costs [16,17]. Soil erosion in Mediterranean olive orchards has been mentioned as one of the significant threats to the sustainability of this crop [17]. On the other hand, the intensive use of chemical fertilizers causes serious environmental hazards as only a small fraction is absorbed. In contrast, a significant fraction is washed up, producing a high accumulation of chemical compounds on surface and ground waters [18].

Current previsions of climate conditions for the next decades in olive-growing areas predict a significant decrease in annual rainfall and an increase in temperature and evapotranspiration, which will threaten this crop even more [17]. Considering the relevance of olive cultivation in the Mediterranean area, it is urgent to implement good soil management practices to maintain good growth conditions, productivity and oil quality to safeguard the olive tree performance, especially during the crucial periods of plant development and fructification [16]. Thus, in the last years, great importance has been given to soil amendments and conditioners. These products that can help regulate the soil biological functions, which may have a relevant role in increasing the sustainability of agricultural systems [15,19,20]. Biochar and zeolites are two of the most widely described.

Biochar is a carbon-rich solid produced by biomass pyrolysis at relatively high temperatures (300–700 °C) [19,20]. The high porosity of this material is one of its unique properties, which can be favourable for improving soil water holding capacity and structure [20]. Furthermore, this substance can modify the physicochemical properties of the soil, promote C sequestration, decrease gaseous N emissions, improve soil nutrient availability, reduce nutrient leaching and increase crop yields [19]. In turn, zeolites are hydrated aluminosilicates of alkali and alkaline earth minerals, and their structure is characterized by a framework of [SiO_4_]^−4^ and [AlO_4_]^−5^ tetrahedron linked to each other by sharing oxygen atoms, forming a three-dimensional framework [20]. This structure confers essential properties, such as a large internal porosity that results in water retention, a uniform particle-size distribution that allows them to be easily incorporated and high cation-exchange capacity that retains nutrients [21,22]. Among the natural zeolites, clinoptilolite is the most abundant and commonly used in agricultural practices [18,21]. One of the main applications in agriculture is their use as an additive to fertilizers, promoting the nutrient-retention capacity of the soils by improving the slower release of these elements for crop uptake [18,23]. Therefore, using zeolites and biochar as soil amendments and slow-release fertilizers is considered a sustainable approach that ameliorates soil physicochemical and biological properties, with a high potential to mitigate climate change [22,24]. However, as far as we know, the effect of biochar and zeolites, used as soil amendments, on olive yield, fruit and oil composition and quality has never been reported. Oil composition and quality vary according to several factors, such as climate conditions, harvest time and agronomic practices [5]. Thus, in addition to search sustainable practices to implement in rainfed olive orchards, it is also important to investigate their effect on the quality of products. The objective of this study was to evaluate the effect of zeolites and biochar on yield, polyphenolic and fat composition and quality parameters of olive fruits and oil obtained from olive orchards managed under rainfed conditions. 

## 2. Materials and Methods

### 2.1. Site Characterization

The field experiment was carried out in 2018 and 2019 in a rainfed olive orchard located at São Pedro Vale do Conde (41°26′36.2″ N 7°13′21.2″ W) in the municipality of Mirandela, northeast of Portugal. The trees of the Cobrançosa cultivar were 18 years old at the beginning of the experiment. Cobrançosa is one of the most important Portuguese cultivars for extra virgin olive oil (EVOO) production [25]. The distance between rows and plants within the rows was 7 × 7 m, with a planting density of 204 trees ha^−1^.

**Figure 1:**
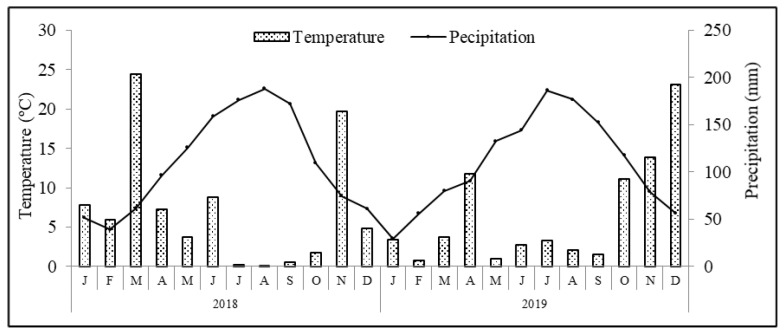
Average monthly temperature and precipitation conditions recorded during the experimental period in the weather station at Paradela close to experimental plot.

The region benefits from a Mediterranean type of climate, where average annual air temperature and accumulated precipitation are 14.3 °C and 509 mm, respectively. Meteorological data recorded during the experimental period in a weather station located at Paradela, close to the experimental plot, are presented in Figure 1. Other climatic variables, such as mean (Tmean), maximum (Tmax) and minimum (Tmin) temperatures, mean temperature from May to October [from blossom to ripeness; Tmean (May–Oct.)], cumulative precipitation (Σ Precp.) and cumulative precipitation from May to October (Σ Precp. (May–October)) are shown in Table 1. The soil of the orchard is a Leptosol sandy-loam texture (74.3% sand, 23.8% silt and 1.9% clay) formed in a schist bedrock. Soil analyses performed before the establishment of the trial revealed an organic carbon content of 4.4 g kg^−1^, pH 5.1, extractable (Egner–Riehm) phosphorus of 18.4 mg kg^−1^ and potassium 43.2 mg kg^−1^, respectively.

### 2.2. Experimental Design

The experiment included three soil treatments, arranged with three replicates and three plants per replication. The treatments corresponded to conventional fertilization (FC), as control, FC plus zeolites (ZL) and FC plus biochar (BC). The conventional fertilization consisted of the annual application of 60 kg of N, P_2_O_5_ and K_2_O ha^−1^ and 2 kg ha^−1^ of B as borax (11% B). Biochar (Ibero Massa Florestal, Oliveira de Azeméis, Portugal) and clinoptilolite zeolites (Zeolita Natural AGRO^®^ (0.6–1.5 mm, ZeoCat, Barcelona, Spain)) are commercial products whose composition is provided in Table 2. These products were only applied in the first year of the experiment, at a rate of 10 t ha^−1^ and 5 t ha^−1^, for biochar and zeolites, respectively. The amendments and fertilizers were applied late in March and were homogeneously spread beneath the tree canopy. No phytosanitary products were used during the experimental period.

### 2.3. Sample Acquisition and Olive Yield

In 2019, samples for soil moisture determination were collected at a depth of 0–20 cm on 3 different summer days: D1 (11 June), D2 (11 July) and D3 (21 August). Soil moisture was determined according to the gravimetric method [26].

Olive trees were harvested on 6 November 2018 and 4 November 2019 with a trunk shaker machine, which detaches the olive fruits and collects them with an associated inverted umbrella system. The yield was weighed per groups of three trees. At the harvest, olive fruit samples were collected for biometric and maturation index (MI) analyses, olive oil extraction and biochemical analysis. Fruit biometric and MI analysis and the olive oil extraction were performed immediately after harvest. In contrast, the remaining olive samples were pitted and stored at −80 °C for posterior biochemical analyses.

### 2.4. Fruit Biometric Variables and Maturation Index

Groups of 50 olives from each treatment and replicate were randomly selected to determine biometric variables of fruit, pulp and pit fresh and dry weight (FW and DW) and longitudinal and equatorial length. The pulp/pit ratio was calculated according to the followed formula:


Pulp/Pit ratio=Pulp FW/Pit FW

MI was determined according to the method proposed by El Yamani et al. [27] through the classification of olive fruits into eight categories based on the epidermis and pulp colour (0–7). The scale starts with fruits with the intense green epidermis (MI = 0) and ends with fruits with black epidermis and totally purple pulp (MI = 7). The MI was calculated as follows:

MI=(a×0+b×1+c×2+d×3+e×4+f×5+g×6+h×7)/n,

where the letters a–h are the number of fruits in each category and n is the total number of olives assessed.

### 2.5. Fruit Fat Content and Fatty Acid Profile Determination

Folch’s extraction method was used [28] with some adaptations to determine the olive fruit fat content and fatty acid profile. Briefly, 50 mL of Folch’s solution (Chloroform:MeOH (3:1) with 75 mg L^−1^ butylated hydroxytoluene) was added to 2 g of lyophilized olive flesh, followed by an ultra-turrax mechanical homogenization. The obtained extract was filtered into a separating funnel, and this step was repeated twice. The volume was adjusted up to 150 mL with Folch’s solution, followed by adding 37.5 mL of NaCl (0.73%). After rest overnight, the organic phase was collected to evaporating flasks and the solvent was completely evaporated on a rotary evaporator at 45 °C. The flask was reweighted, after 24 h in a desiccator. The fat content was calculated as follows: Fat content (%)=((W1−W0))/Ws×100, where W1 is the flask weight after evaporation, W0 is the initial flask weight and Ws is the initial sample weight. The evaporated content was diluted in 2 mL of *n*-hexane and submitted to a derivatization procedure to promote the conversion of the free fatty acids to their methyl esters. For the derivatization, 100 µL of lipid extract was added to 2 mL of MeOH:*n*-hexane (2:1) and placed on ice with the careful addition of 200 µL acetyl chloride. After 1 h at 100 °C on a heating block, 1.5 mL of *n*-hexane and 6 mL of potassium carbonate 6% were added. Then, the mixture was centrifuged at 2864 rpm for 5 min. The organic phase of each sample was collected and used for the chromatographic analysis on a Trace GC gas chromatograph equipped with a flame ionized detector and Autosampler, fitted with a fused silica capillary column (Supelcowax^®^ 10, with 30 m length × 0.25 mm ID and 0.25 µm film thickness). For the chromatographic analysis, 1 µL of each sample was injected and submitted to a total run of 48 min, programmed to start with an oven temperature of 140 °C during 2 min, followed by a gradient from 140 °C to 220 °C (4 °C/min) and maintained at 220 °C for 20 min. The injector (splitless) and detector temperatures were held at 250 °C. Helium was used as the carrier gas at a flow rate of 1 mL/min. All samples were run in triplicate, and the results were expressed in relative percentage for each fatty acid, calculated by internal normalization of the chromatographic peak area. The fatty acid profile analysis was performed using XCalibur TM Software (Thermo Fisher Scientific, Waltham, MA, USA) by comparing the retention times (RT) of the sample’s peaks with those from reference standard run on the same conditions. The sample’s fatty acid profile was presented in terms of SFAs: myristic acid (C14:0), palmitic (C16:0), arachidic (C20:0), behenic (C22:0) and unsaturated acids: MUFAs—palmitoleic (C16:1), oleic (C18:1), gondoic acid (C20:1), erucic acid (C22:1), and PUFAs—linoleic (C18:2) and linolenic (C18:3).

### 2.6. Olive Oil Extraction and Quality Analyses

The olive oil extraction was performed immediately after the fruit harvest. The oil was extracted by processing 20 kg of olives at the malaxation temperature of 25 °C for 30 min, using olive oil extraction equipment (OLIOMIO 50, Toscana Enologica Mori, Tavarnelle Val di Pesa, Italy). After that, oils were filtered and placed in dark glass bottles and kept at 4 °C, analysed within two days after oil extraction.

The olive oil quality parameters of free acidity (FA), peroxide index (PI), K232, K270 and ΔK were determined according to the European Community Regulation EEC/2568/91 [29]. FA was expressed as % oleic acid per 100 g of olive oil and PI as mEq of O_2_ kg^−1^ of oil.

### 2.7. Extraction and Quantification of Polyphenolic Compounds from Olive Fruits and Olive Oil

To extract polyphenolic compounds from olive fruits, 30 mL of MeOH:H_2_O (50:50) were added to 2 g of lyophilized olive flesh. After shaking for 30 min at room temperature, the samples were centrifuged at 2988 rpm for 10 min. The methanolic extract resultant from centrifugation was reserved, and this step was repeated three times. To remove the fat phase of the final extract, we added 50 mL of *n*-hexane twice, and the organic phase was discarded. The volume was adjusted to 200 mL with MeOH:H_2_O (50:50). For olive oil polyphenolic extraction, 3 mL of oil were used along with 1.25 mL MeOH:H_2_O (70:30) and 1.25 mL *n*-hexane. The mixture was centrifuged for 10 min at 5000 rpm. The lower phase was carefully collected. This procedure was repeated three times. The final extract was adjusted to 5 mL with MeOH/H_2_O (70:30). The obtained extracts were used for the quantification of total phenols (TP), ortho-diphenols, flavonoids and total antioxidant activity (TAC), which were performed as described by Brito et al. [30]. TP and ortho-diphenols were expressed as mg of gallic acid equivalents (GAE) and flavonoids were expressed as mg of catechin equivalents (CE) per g of olive flesh DW or kg of olive oil. TAC was expressed as mmol of Trolox Equivalent (TE) per g of olive flesh DW or kg of oil. All measurements were performed in triplicate.

### 2.8. High-Performance Liquid Chromatography (HPLC) Analysis of Olive Fruits and Oil Polyphenols

For olive fruit HPLC analysis, 100 mL of olive flesh methanolic extract were evaporated at 35 °C and redissolved in 2 mL of MeOH:H_2_O (50:50). The phenolic profile was performed by reversed-phase (C18) HPLC using an Ultimate 3000 HPLC system (Dionex Corporation, Sunnyvale, CA, USA), equipped with an Ultimate 3000 pump and column compartment, a WPS-3000 TSL analytics auto sampler and a photodiode array detector (PDA-100, Dionex Corporation, Sunnyvale, CA, USA). The compounds separation was reached by gradient elution on an ACE 5 C-18 column (250 × 4.6 mm) (Advanced Chromatography Technologies, Scotland). The eluent was constituted of 0.1% aqueous formic acid (solvent A) and methanol (solvent B). The elution program was characterized by a linear gradient analysis for a total run time of 80 min used as follows: 5% of solvent B during 2 min, increased to 80% solvent B over 68 min, isocratic for 8 min, decreasing to 5% solvent B over 2 min, and last isocratic for 5 min. The photodiode detector was operated between 200–600 nm and the chromatographic profile was recorded at 280 and 325 nm. The sample volume injected was 50 µL at a flow rate of 0.5 mL min^−1^, and the column temperature was maintained at 30 °C.

Quantification was performed with calibration curves with standard (−)gallocatechin, hydroxytyrosol, tyrosol, (−)epigallocatechin, chlorogenic acid, epicatechin, quercetin-3.7-di-*O*-glucoside, luteolin-3.7-di-*O*-glucoside, apigenin-7-*O*-glucoside, verbascoside, oleuropein, rutin and apigenin.

For olive oil polyphenolic HPLC analysis, 5 mL of olive oil methanolic extract was evaporated at 35 °C and redissolved in 2 mL of MeOH:H_2_O (70:30). The phenolic profile was analysed by a Thermo Fisher Scientific Vanquish Core HPLC system (Waltham, MA, USA), equipped with a pump, column compartment, an auto sampler and a diode array detector. The compounds separation was reached by gradient elution on a C18 Merck Purospher^®^ STAR, Hibar^®^ C18 column (250 × 4.6 mm; particle size 5 μm). The eluent was constituted by 0.1% aqueous formic acid (solvent A) and methanol (solvent B). The elution program was characterized by a linear gradient analysis for a total run time of 100 min used as follows: initiates with 20% of solvent B, followed by an increase to 95% solvent B over 90 min, isocratic for 5 min, decreasing to 20% solvent B over 1 min, and last isocratic for 4 min. The photodiode detector was operated between 200–600 nm and the chromatographic profile was recorded at 280 and 325 nm. The sample volume injected was 50 µL at a flow rate of 0.5 mL min^−1^, and the column temperature was maintained at 30 °C. Quantification was performed based on calibration curves of hydroxytyrosol, verbascoside, luteolin-7-*O*-glucoside, rutin, apigenin-7-*O*-glucoside, oleuropein, luteolin and apigenin. All standards were purchased from Sigma-Aldrich (Burlington, MA, USA). Calibration curves were prepared in a concentration range of 5–500 mg L^−1^ using each phenolic compound’s maximum absorption wavelength. Hydroxytyrosol calibration curve was used to quantify 3,4-Dihydroxyphenylglycol (DHPG), tyrosol, hydroxytyrosol acetate and dialdehydic form of decarboxymethyl elenolic acid linked to hydroxytyrosol (3,4-DHPEA-EDA). Calibration curve of oleuropein was used for the quantification of oleuropein aglycon. These last compounds were identified according to Kanakis et al. [31] and Tasioula-Margari and Tsabolatidou [32]. Data acquisition, analysis and peak integration were performed using Chromeleon software (Version 7.1; Dionex, Thermo Fisher Scientific, Waltham, MA, USA).

### 2.9. Statistical Analysis

Statistically significant differences between means were determined by analysis of variance (one-way and two-way ANOVA) followed by Tukey’s honestly significant difference (HSD, 5% level) post hoc test. One-way ANOVA was performed to assess the differences between treatments in each year. A two-way ANOVA was performed to evaluate the differences obtained by treatment, year and interaction. These analyses were performed using the JMP statistical software v. Pro 14 (SAS Institute Inc., Cary, NC, USA). To evaluate the relationship between olive fruit and oil phenolic compounds and fatty acids, an orthogonal partial least squares-discriminant analysis (OPLS-DA) was performed using the SIMCA software v. 14.1 (Umetrics, Umea, Sweden). Multiple Factor analysis (MFA) of the fruit and oil phenolic compounds data was performed using XLSTAT (Addinsoft, Anglesey, UK).

## 3. Results and Discussion

### 3.1. Climate Data and Soil Moisture

Climate conditions verified during the experimental period followed the typical patterns of a Mediterranean climate, with generally hot and dry weather from May to September, and it being mild and wet the rest of the year. The total rainfall was 708.4 and 652.2 mm in 2018 and 2019, respectively (Table 1). The average annual temperature was 13.2 °C in 2018 and 13.0 °C in 2019. Concerning the registered temperatures and precipitation patterns, differences between years were found. In particular, in 2018, the average rainfall from blossom to ripening (May–October) was 125.8 mm, with an average temperature of 18.6 °C. In contrast, in 2019, the average rainfall was 179.8 mm with an average temperature of 18.2 °C.

**Figure 2:**
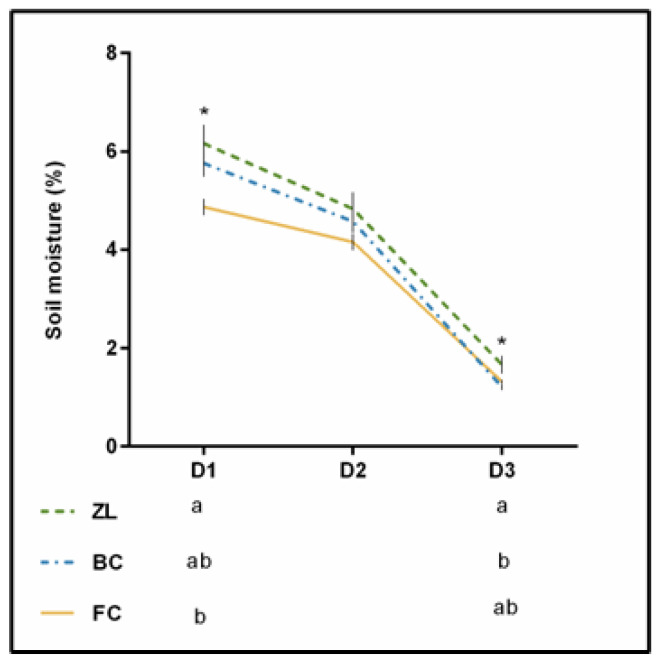
Soil moisture (%) recorded during 3 dates during the summer of 2019. * means significant difference at *p* < 0.05.

Figure 2 presents the soil moisture obtained on three sampling days in the summer of 2019. Differences were statistically significant at D1 and D3, with the ZL plot showing the highest soil moisture. It has been reported that natural zeolites may influence the soil structure and its general quality due to their chemical and physical properties. The high internal pore volume can efficiently improve water holding capacity. Furthermore, the open network of the zeolites structure can lead to new routes for water movement, which can consequently enhance infiltration rate and saturated hydraulic conductivity [18,24]. Thus, zeolites can improve the water content of treated soils [33].

### 3.2. Effect of Soil Treatments on Olive Yield, Fruit Biometric Variables and Maturation Index

Olive yield obtained during the experimental period is presented in Table 3. Applying zeolites and biochar had no significant effect on crop yield in both years. Similarly, a lack of a substantial impact on crop yield by the short-term use of zeolites and biochar was reported by some authors [34,35,36]. Haider [37] showed that biochar needs a certain degree of ageing in the soil to affect the crop yield positively. It may be due to the slow development of an organic coating on the biochar surface after the aging in compost media, which enhances nutrient retention [36]. In turn, using natural zeolites in combination with chemical fertilizers may act as a slow-release fertilizer, allowing the gradual release of nutrients [18]. This may be why there was no positive response to soil conditioners in the olive yield in a short-term assessment. These observations substantiate the requirement of long-term soil amendments application field trials to accurately analyse its effects on crop yield and soil quality [36].

The effects of soil treatments on the biometric variables of fruits obtained from the harvests of 2018 and 2019 are also shown in Table 3. The results varied according to soil treatment and harvest year. In 2018, both FC and BC treatments registered higher values of fruit FW, equatorial and longitudinal length. In addition, in 2019, ZL presented higher values of pulp/pit ratio than BC treatment. Fruit MI was generally higher in 2019 than in 2018. In 2018, MI was 3.33 for FC and ZL and 3.47 for BC, while in 2019, MI was 3.13 for FC, 4.00 for ZL and 3.67 for BC. These differences may be due to alternate bearing, a frequent phenomenon in the olive tree, leading to alternately low and high yields yearly. Although without significant differences for the other treatments, ZL had the highest average olive yield in 2018 and the lowest in 2019. In the olive tree, higher yields result in smaller fruit and delayed ripening and vice versa. This may justify the differences in biometric variables observed between years.

### 3.3. Olive Fruit and Oil Metabolite Concentration under the Influence of Soil Conditioners

The content of ortho-diphenols, flavonoids and TAC obtained in olives and olive oil is shown in Table 4. The results showed fluctuations between soil treatments and years, without a clear tendency. In 2018, olive fruits from both FC and ZL treatments showed significantly higher content of ortho-diphenols and TAC. Interestingly, this trend was reversed in the olive oil obtained in the same year, as BC treatment presented higher levels of ortho-diphenols, flavonoids and TAC. In 2019, BC fruits presented significantly higher content of ortho-diphenols and flavonoids and lower TAC than the other treatments. Regarding olive oil, ZL presented a significantly higher concentration of ortho-diphenols, flavonoids and TAC, while BC olive oil showed higher flavonoids content. According to these data, only a tendency for the consecutive accumulation of flavonoids on BC olive oils was observed. The biosynthesis and accumulation of flavonoids is a well-recognized adaptive mechanism in plants to cope with several biotic and abiotic stresses due to its high antioxidant capacity [38]. Their accumulation in olive oil promotes high antioxidant, anti-inflammatory, antimicrobial and antitumoral activity [5]. In 2019, BC treatment showed low soil moisture during the summer, presenting the lowest levels of D3 (August). This may have induced the biosynthesis and accumulation of flavonoids, to increase the antioxidant capacity.

Significant differences between treatments were observed regarding the influence of the harvest year on the accumulation of fruit and oil metabolites. Both olive fruits and oils from 2019 presented higher flavonoids and TAC content and lower ortho-diphenols content than in 2018. As previously mentioned, accumulating phenolic compounds, particularly flavonoids, is considered a protective response to drought stress. Although 2018 registered more drought conditions from flowering to fruit ripeness, the trend of flavonoid accumulation was not verified, presenting only higher content of ortho-diphenols. However, the reduced TAC in olive fruits and oil in 2018 indicates stress conditions. Such imposed stress is commonly accompanied by an increase in reactive oxygen species production that leads to an imbalance between their production and scavenging, and a reduction in antioxidant activity [39].

### 3.4. Influence of Soil Amendments on Olive Fruit and Oil Polyphenolic Composition

The olive fruit polyphenolic composition is presented in Table 5. The chromatographic analysis identified a total of 13 compounds. All samples showed a similar chromatographic profile, registering only variations in polyphenolic concentration between treatments. According to Vinha et al. [40], the most important phenolic compounds in olive fruit include secoiridoids, flavonoids, phenolic acids, phenolic alcohols and hydroxycinnamic acids. The most predominant secoiridoid of olive fruits is oleuropein, which confers important nutritional and sensorial properties, as well as resistance against autoxidation and photoxidation. Moreover, the most frequent flavonoids are rutin, luteolin and apigenin; the most common phenolic alcohols are hydroxytyrosol and tyrosol and verbascoside is the principal hydroxycinnamic acid derivative [40]. In the present study, the major phenolic compounds found in the olive fruit samples were oleuropein, luteolin-3,7-di-*O*-glucoside, rutin, hydroxytyrosol and verbascoside, which is in accordance with the study mentioned above.

Several factors can influence the concentration of phenolic compounds, such as geographical zone, agro-climatic conditions and degree of fruit ripeness among others [14,41]. The results obtained in this study varied according to soil treatment and harvest year. In 2018, olive fruits from ZL treatment showed the highest content of total phenolics obtained by the sum of the individual phenolic compounds (TP), which was due to the higher content of tyrosol, chlorogenic acid, verbascoside, oleuropein, epicatechin, rutin and apigenin-7-*O*-glucoside. In turn, FC fruits showed higher levels of chlorogenic acid, epigallocatechin and quercetin-3,7-di-*O*-glucoside, and BC fruits presented superior concentrations of tyrosol, apigenin-7-*O*-glucoside and apigenin. In 2019, a higher content of TP was obtained in olive fruits from FC, due to the increase in hydroxytyrosol, tyrosol, verbascoside, oleuropein, gallocatechin, epigallocatechin and rutin. ZL fruits showed an increase in chlorogenic acid, quercetin-3,7-di-*O*-glucoside, luteolin-3,7-di-*O*-glucoside and rutin, while BC fruits showed an increase in tyrosol, epigallocatechin, epicatechin, quercetin-3,7-di-*O*-glucoside, luteolin-3,7-di-*O*-glucoside and apigenin-7-*O*-glucoside.

Despite the differences between treatments and harvest years, a trend was observed in both the years of accumulation of tyrosol and apigenin-7-*O*-glucoside on BC olive fruits, and the accumulation of chlorogenic acid, quercetin-3,7-di-*O*-glucoside and rutin on ZL olive fruits. In addition, BC fruits showed a lower content of TP during the two harvest years. It was previously mentioned that in 2019 the application of BC increased the accumulation of olive fruit flavonoid compounds, probably due to facing the water stress promoted by the lower soil moisture. However, this increase in flavonoids was not reflected in the TP content. Due to its properties, biochar may favour plant growth conditions by enhancing soil nutrient availability, plant nutrient uptake, water retention, and soil C:N ratio, thereby affecting the content of secondary metabolites [41,42]. Furthermore, it has been demonstrated that higher soil nutrient availability has a negative correlation with the content of polyphenols once the low concentration of certain minerals in the soil, such as N, Ca, Mn, Fe and Zn, stimulates the phenylpropanoid metabolism, and particularly inducing the accumulation of phenylalanine ammonia-lyase (PAL), the first enzyme of the pathway [20,43]. Thus, the lower phenolic compound accumulation observed on BC olive fruits was probably associated with a higher soil nutrient availability and plant uptake, promoted by the application of biochar and chemical fertilizer, as reported by Ding et al. [19].

**Figure 3:**
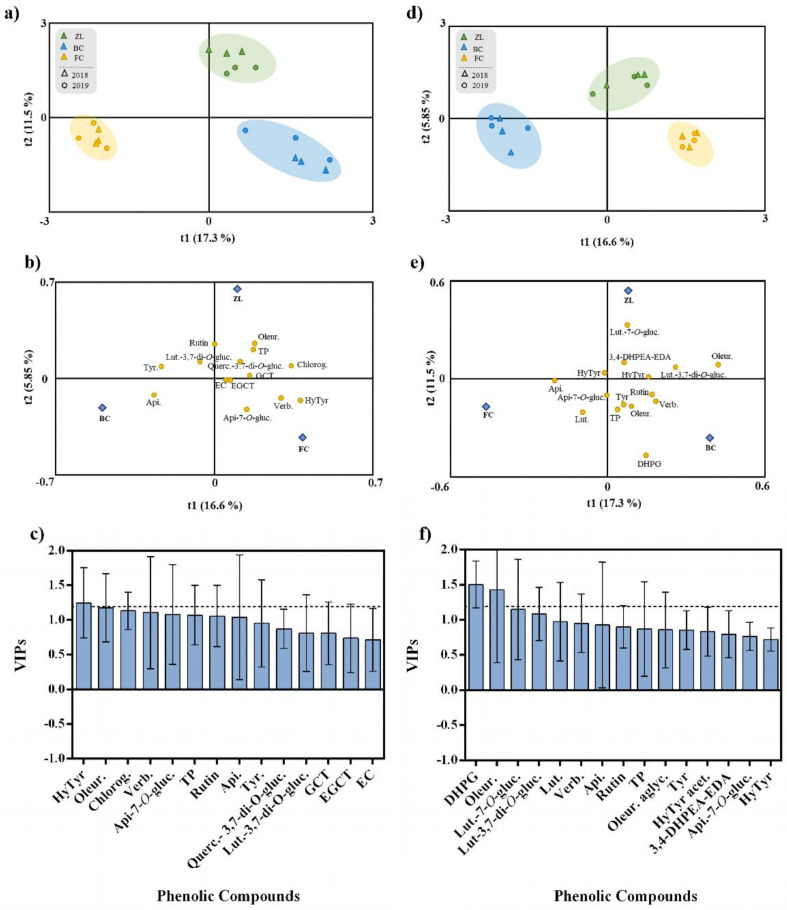
OPLS-DA of olive fruit (**a**–**c**) and olive oil (**d**–**f**) phenolic compounds, irrespective to the year. Scores plot (**a**,**d**) and loadings plot (b and d) of the first two factors of the OPLS-DA model built with the phenolic profile of the olive oil according to the soil treatment. Phenolic compounds ranked by VIP scores (**c**,**f**). Hydroxytyrosol (HyTyr), oleuropein (Oleur.), chlorogenic acid (Chlorog.), verbascoside (Verb.), apigenin-7-*O*-glucoside (Api-7-O-gluc.), total phenols (TP), apigenin (Api.), tyrosol (Tyr), quercetin-3,7-di-*O*-glucoside (Querc.), luteolin-3,7-di-O-glucoside (Lut-3,7-di-*O*-gluc.), gallocatechin (GCT), epigallocatechin (EGCT), epicatechin (EC),3,4-dihydroxyphenylglycol (DHPG), luteolin-7-*O*-glucuside (Lut-7-*O*-gluc.), luteolin (Lut.), oleuropein aglycone (Oleur. aglyc.), hydroxytyrosol acetate (HyTyr acet.) and dialdehydic forms of decarboxymethyl elenolic acid linked to hydroxytyrosol (3,4-DHPEA-EDA).

To explore the effect of the different soil treatments on olive fruit and oil phenolic composition, irrespective of the harvest year, we performed an OPLS-DA. This analysis represents a more straightforward and easier interpretation of the models, focusing on the predictive, i.e., discriminant information summarised in the predictive components. The fitting parameter (R2) and the predictive ability (Q2) were utilized to evaluate the quality of the models [44]. The OPLS-DA respective to the olive fruits are presented in Figure 3a–c. The class prediction model discriminated the olive fruits according to soil treatments, confirming that the actual phenolic profile of olive fruits was modulated by the soil conditioner treatments (Figure 3a). This result supports the statement that agronomic practices and soil properties influence the fruit polyphenolic composition [5]. The discrimination between treatments was due to the positive correlation with some specific phenolic compounds. According to Figure 3b, FC olive fruits were highly correlated with verbascoside, apigenin-7-*O*-glucoside and hydroxytyrosol, and ZL fruits were correlated with oleuropein, TP, chlorogenic acid and quercetin-3,7-di-*O*-glucoside. In contrast, BC olive fruits were correlated with apigenin, tyrosol and luteolin-3,7-di-*O*-glucoside. The OPLS-DA quality parameters of R2Y and Q2Y were 0.93 and 0.77, respectively. No outlier samples could be observed considering the model Hotelling’s T2. The CV-ANOVA and permutation tests, given as supplementary results (Table S1 and Figure S1), showed an adequate degree of validation. Afterwards, the variables importance in the projection of the OPLS-DA model was evaluated by variable importance (VIP) analysis, particularly considering the VIP scores for each phenolic compound analysed. The VIP score summarizes each variable’s impact on the model, and it is calculated as a weighted sum of the squared correlations between the OPLS-DA components and the original variables. Olive fruit phenolic compounds with the highest VIP scores (>1.2) were hydroxytyrosol and oleuropein (Figure 3c).

Table 6 summarizes the polyphenolic composition of olive oil. A total of 14 phenolic compounds were identified by the chromatographic analysis, 8 of them common to olive fruits. The main process steps involved in olive oil extraction consist of cleaning harvested olives, crushing, malaxing and phase separation. The proportion and profile of phenolics in olive oil partly reflects the rate of their solubilization and chemical reactions, which are highly affected by the processing conditions. These chemical alterations, mainly due to enzymatic activity, occur when the olive paste is in contact with the air during the malaxation process, which may adversely develop degradation/oxidation of aglycones [45]; in particular oleuropein and demethyloleuropein are hydrolysed by endogenous β-glycosidases to 3,4-DHPEA-EDA and oleuropein aglycone (3,4-DHPEA-EA). These newly formed substances are the most abundant secoiridoids in olive oil [46]. Secoiridoids are insoluble in oil; therefore, only a small percentage of these compounds are present in olive oil after the mechanical extraction process. Nevertheless, they are one of the most important compounds on EVOO for their sensorial and health properties [5]. In addition, among the major phenolics found in olive oil, tyrosol and hydroxytyrosol, as well as their derivatives, are the main phenolic alcohols, and luteolin, apigenin and their derivatives are the principal flavonoids [5].

In the present study, the major phenolic compounds identified in olive oil samples were oleuropein aglycone, luteolin and apigenin. According to literature, olive oil TP content ranges between 50 and 1000 mg kg^−1^, being more common in concentrations between 100 and 300 mg kg^−1^ [5]. Our results showed a lower olive oil TP content, with values ranging between 44.2–64.5 mg kg^−1^; 2018 was the year where the highest values were recorded. Similar to what happens with olive fruits, several factors may influence the content of polyphenols in olive oils, such as olive cultivar, environmental conditions, agronomic practices, maturation stage and extraction conditions [45]. Our results showed significant differences between soil treatments and harvest years. In 2018, the olive oil obtained from BC treatment presented a higher content of TP as a consequence of the increase in DHPG, tyrosol, verbascoside, oleuropein, rutin, apigenin-7-*O*-glucoside and luteolin. In turn, ZL olive oil showed a higher concentration of hydroxytyrosol, 3,4-DHPEA-EDA, oleuropein, luteolin-7-*O*-glucoside and apigenin, while FC showed the lowest content of almost all phenolic compounds, and consequently the lowest TP. Contrarily, in 2019, olive oil obtained from FC treatment presented a higher content of TP, as a consequence of the increase in apigenin-7-*O*-glucoside, luteolin and apigenin. Biochar treatment promoted an increase in DHPG, hydroxytyrosol acetate, oleuropein aglycone, 3,4-DHPEA-EDA, oleuropein and rutin on olive oil, while ZL showed an increase only on luteolin-3,7-di-*O*-glucoside.

The tendency to low levels of TP observed in olive fruits from biochar plot during both years was not verified in olive oil, as ZL olive oils showed low levels of TP. It was already mentioned that this treatment conferred a higher soil water availability, which may have promoted a better plant protective status, and, therefore, a lower necessity of investment on secondary metabolites, which was reflected in olive oil polyphenolic composition. In turn, BC olive oils showed the consequent accumulation of some phenols with huge nutritional value, such as DHPG, oleuropein and rutin. Nutraceutical properties have been attributed to oleuropein, hydroxytyrosol and their derivatives, as is the case of DHPG, due to its high potential as antioxidant agents [47].

In the same way, an OPLS-DA was performed to analyse the effect of the different soil amendments on olive oil polyphenolic composition, irrespective of the harvest year (Figure 3d–f). The class prediction model discriminated the olive oils according to the different soil treatments (Figure 3d). Similar to what happened with olive fruits, these results confirmed that the different soil conditioners imposed the phenolic profile of olive oil. In this regard, the quality parameters of the OPLS-DA model were excellent with R2Y and Q2Y very high (being 0.94 and 0.91, respectively). According to Hotelling’s T2 analysis, no outlier samples were observed. The analyses of CV-ANOVA (Table S2) and permutation tests (Figure S2), given as supplementary results, showed more than the adequate degree of validation. According to Figure 3e, the compounds positively correlated with FC olive oil were apigenin, apigenin-7-*O*-glucoside and luteolin, while ZL olive oil was highly correlated with luteolin-7-*O*-glucoside, 3,4-DHPEA-EDA, hydroxytyrosol acetate and luteolin-3,7-di-*O*-glucoside, and BC olive oil was correlated with DHPG, TP, oleuropein, tyrosol, rutin and verbascoside. Moreover, phenolic compounds with the highest VIP score (>1.2) were DHPG and oleuropein (Figure 3f).

**Figure 4:**
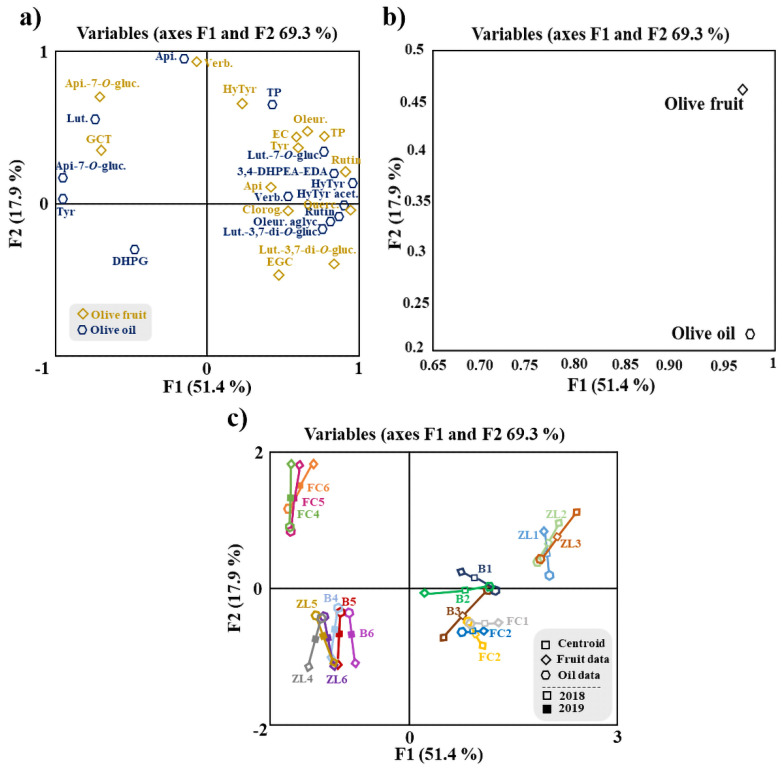
MFA of phenolic compounds of olive fruit and olive oil. (**a**) Representation of olive samples and clouds; (**b**) representation of groups (tables) of variables; (**c**) distribution of variables. Centroid (☐); olive fruit data (◆); olive oil data (⬟); centroids respective to samples of 2018 (☐) and 2019 (■). Apigenin-7-*O*-glucoside (Api-7-*O*-gluc.), luteolin-7-*O*-glucuside (Lut-7-*O*-gluc.), luteolin-3,7-di-*O*-glucoside (Lut.-3,7-di-*O*-gluc.), verbascoside (Verb.), 3,4-dihydroxyphenylglycol (DHPG), oleuropein aglycone (Oleur. aglyc.), tyrosol (Tyr), dialdehydic forms of decarboxymethyl elenolic acid linked to hydroxytyrosol (3,4-DHPEA-EDA), hydroxytyrosol (HyTyr), apigenin (Api.), total phenols (TP), oleuropein (Oleur.), luteolin (Lut.), hydroxytyrosol acetate (HyTyr acet.), epigallocatechin (ECGT), epicatechin (EC), gallocatechin (GCT), chlorogenic acid (Chlorog.) and quercetin−3,7-di-*O*-glucoside (Querc.).

Additionally, an MFA was applied to the data of fruit and oil phenolic compounds (Figure 4). MFA is a factorial method devoted to studying of tables in which a group of individuals is described by a set of variables (quantitative and/or qualitative) structured in groups. Figure 4a shows the olive fruit and oil samples and clouds. The coordinates of the variables (tables) were displayed and used to create the map of tables (Figure 4b). Considering the variables map, it can be concluded that both groups contributed almost equally for the first factor (96.9% for the fruit and 97.5% for the oil), while for the second factor, fruit variables contributed 46.2% and oil variables 21.7%. For the Lg measurements, the first axis corresponded to 1.143 for the olive fruit variable and 1.025 for the olive oil variable. The olive fruit variable was positively correlated with F1 and F2. This loading can be related to hydroxytyrosol, oleuropein, epicatechin, TP, tyrosol, rutin and apigenin. In contrast, the olive oil variable is positively correlated only with F1, mainly associated with rutin, oleuropein aglycone and luteolin-3,7-di-*O*-glucoside. The phenolic compounds correlated with the olive fruit variable are in agreement with the literature, since they are the major polyphenol constituents found in olives, while among the phenolic compounds most correlated with the variable olive oil, only oleuropein aglycone has been described as a major phenolic constituent of oil [5,14].

**Figure 5:**
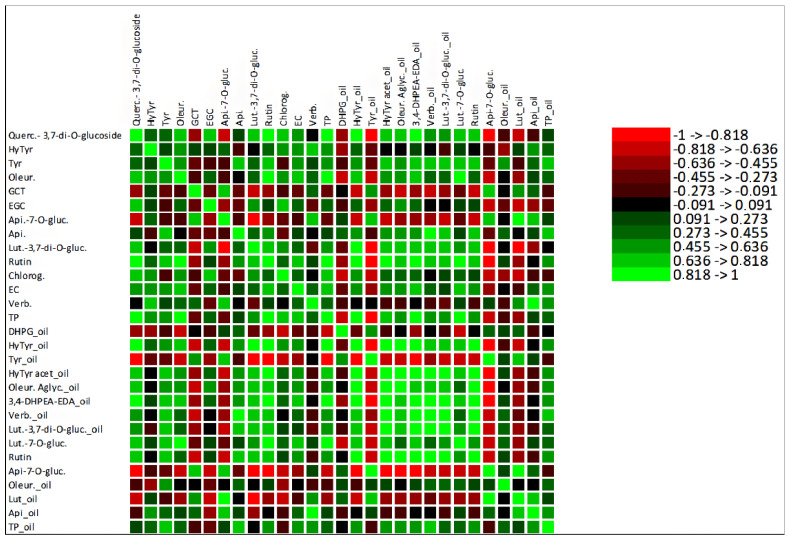
Heatmap of the correlation matrix between olive fruit phenolic compounds and olive oil phenolic compounds. Quercetin-3,7-di-*O*-glucoside (Querc-3,7-di-*O*-glucoside), hydroxytyrosol (HyTyr), tyrosol (Tyr), oleuropein (Oleur.), gallocatechin (GCT), epigallocatechin (ECG), apigenin-7-*O*-glucoside (Api-7-*O*-gluc.), apigenin (Api.), luteolin-3,7-di-*O*-glucuside (Lut-3,7-di-*O*-gluc.), chlorogenic acid (Chlorog.), epicatechin (EC), verbascoside (Verb.), total phenols (TP), 3,4-dihydroxyphenylglycol (DHPG), hydroxytyrosol acetate (HyTyr acet.), oleuropein aglycone (Oleur. aglyc.), dialdehydic forms of decarboxymethyl elenolic acid linked to hydroxytyrosol (3,4-DHPEA-EDA), luteolin-7-*O*-glucuside (Lut-7-*O*-gluc.) and luteolin (Lut.).

A correlation analysis was performed to explore the contribution of the total amount of olive fruit phenolic compounds to the olive oil total phenolic compounds, shown in supplementary results (Figure S3). Despite the weak correlation between the TP of these samples, with an R^2^ of 0.1742, the MFA (Figure 4a) showed strong correlations between some olive fruit and olive oil polyphenols, which was also confirmed by the Pearson correlation analysis. Among these correlations stands out the positive correlation between tyrosol from olive fruit and olive oil polyphenols 3,4-DHPEA-EDA, verbascoside and TP. Olive fruit oleuropein was strongly correlated with olive oil DHPG, hydroxytyrosol, tyrosol, 3,4-DHPEA-EDA and luteolin-7-*O*-glucoside, and rutin from olive fruit was highly correlated with hydroxytyrosol, tyrosol, hydroxytyrosol acetate, 3,4-DHPEA-EDA and luteolin-7-*O*-glucoside from olive oil. These strong correlations are explained by the fact that all the polyphenols mentioned above are biosynthetically related, belonging to the same metabolic pathway. Thus, tyrosol may be converted into oleuropein, 3,4-DHPEA-EDA, oleuropein aglycone, hydroxytyrosol and its derivatives. Hydroxytyrosol may be converted into verbascoside and tyrosol [48]. Moreover, the analysis shows that the olive fruit polyphenols more correlated with olive oil TP were tyrosol, apigenin, epicatechin and verbascoside. The detailed correlations between olive fruit and olive oil phenolic compounds are presented in a correlation matrix (Figure 5).

### 3.5. Olive Fruit Fat Content and Fatty Acid Profile under the Influence of Different Soil Conditioners

Table 7 summarizes the results of fruit fat content and fatty acid profile. It has been described that factors, such as temperature, water availability and ripening stage, affect considerably the fat content and fatty acid composition of olive fruits [5]. Early fruit ripening stages are associated with the highest fat content [49]. This agrees with the results obtained in 2019, in which ZL fruits presented a lower fat content and were accompanied by a more advanced maturation stage compared to the other treatments. Regarding results of fruit fatty acid composition, the fatty acid obtained in major concentration was oleic acid (C18:1) (ranging between 72.0% and 76.7%), followed by the palmitic acid (C16:0) (ranging between 12.5% to 14.4%), linoleic acid (C18:3) (ranging between 7.67% to 9.41%), palmitoleic acid (C16:1) (ranging between 0.724% and 1.03%) and linolenic acid (C18:3) (ranging between 0.549% and 0.711%). The obtained values are within the defined thresholds for each fatty acid, namely, 55.0–83.0% for oleic acid, 7.5–20.0% for palmitic acid, 3.5–21.0% for linoleic acid, 0.3–3.5% for palmitoleic acid and 0–1.5% for linolenic acid [50].

In 2018, olive fruits of FC and BC treatments presented higher content of PUFA due to the higher linoleic acid content. ZL olive fruits showed a significantly higher MUFA and oleic/linoleic ratio due to the significantly higher oleic acid content. On the other hand, in 2019, FC fruits presented significantly higher SFA due to an increase in palmitic acid, ZL fruits showed an increase in SFA and PUFA, promoted by an increase in palmitic acid and linolenic acid, respectively, and BC fruits showed higher MUFA due to the increased oleic acid concentration.

Fatty acid composition is important for the commercial properties of oils. In particular, high levels of MUFA result in a wide range of health benefits, such as improved cholesterol levels and, in turn, the prevention of cardiovascular diseases [51]. In addition, the MUFA oleic acid confers important properties of oil stability. Contrarily, the increase in PUFA had a negative influence on the stability of oils due to their contribution to oil rancidity. Therefore, the ratio oleic/linoleic has great importance because of the effects on the nutritional properties and oxidative stability of olive oils [49]. Thus, according to the obtained results, olive fruits with better nutritional and stability properties were from ZL in 2018, considering the increase in MUFA and decrease of PUFA, and, consequently, the higher oleic/linoleic ratio. Although BC fruits from 2019 showed higher MUFA content, no significant differences were observed in PUFA content, which did not impact the oleic/linoleic ratio.

Relative to the differences between harvest years, the year 2018 registered significant higher levels of SFA and PUFA, and a lower value of MUFA, which was due to an increase in palmitic, palmitoleic and linoleic acids and a decrease in oleic acid. It is known that water availability during the fruit development stages negatively affects olive oil’s nutritional value, especially by elevating SFA and decreasing the levels of MUFAs [52]. As previously mentioned, the year 2018 faced an increase in average temperatures and a decrease in rainfall (Table S2), which may explain the differences in fruit fatty acid profile between years.

**Figure 6:**
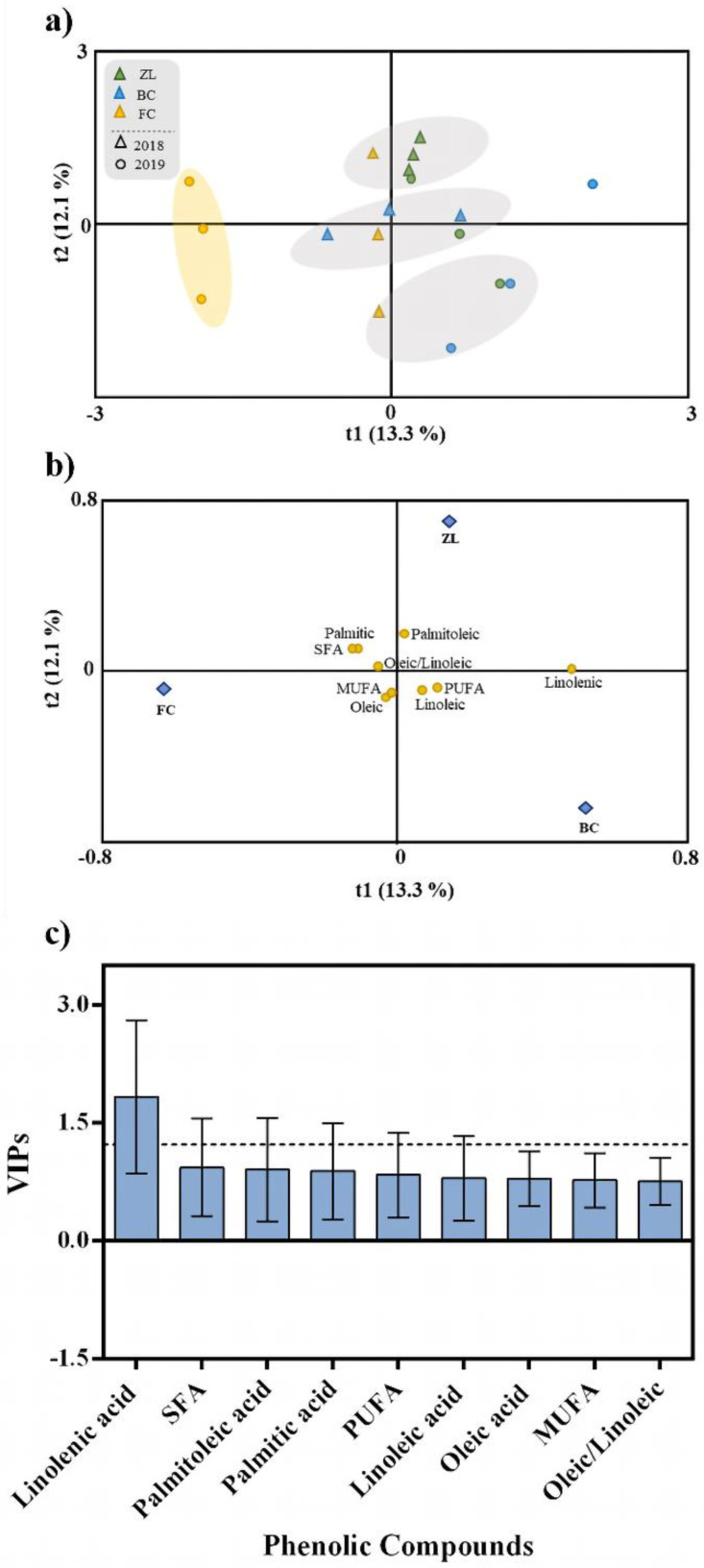
Scores plot (**a**) and loadings plot (**b**) of the first two factors of the OPLS-DA model built with the fruit fatty acid profile according to the soil treatment. Fatty acids ranked by VIP scores (**c**).

In the same way as for phenolic compounds, an OPLS-DA was performed to investigate the influence of the different soil conditioners on olive fruit fatty acid composition (Figure 6). According to the OPLS-DA and the cluster analyses, samples were distributed in several groups and organized independently of the soil treatment, except for the olive samples belonging to the FC treatment from 2019 (Figure 6a). Unlike olive fruit and oil polyphenols, soil treatments did not influence fruit fatty acid composition. This was confirmed by the low-quality parameters of the OPLS-DA model, with values of 0.331 for R2Y and 0.059 for Q2Y. The CV-ANOVA and the permutation test respective to this analysis are in the supplementary material (Table S3 and Figure S4). According to VIP analysis (Figure 6c), the only variable that contributed to the discrimination was linolenic acid (VIP > 1.2).

### 3.6. Impact of Soil Conditioners on Olive Oil Physico-Chemical Variables

Depending on the quality and resultant organoleptic characteristics, the EU defined, in Article 118 of European Commission (EC) Regulation No 1234/2007, six main types of oils, which are also delineated by the International Olive Council (IOC) [53]. These categories are virgin olive oil (VOO), refined olive oil, olive oil composed of refined olive oils and virgin olive oils, crude olive pomace oil, refined olive pomace oil and olive pomace oil [5,53]. Among VOOs, the EU establishes three types of oils: EVOO, VOO and lampante [5]. EVOO is considered to have the highest quality, followed by VOO, which is of good quality, while lampante is characterized as VOO not fit for consumption. This categorization is based on several quality indicators, such as FA, PI and ultraviolet absorption coefficients, including K232, K270 and ΔK. In detail, FA measure the release of the fatty acid chains indicating the hydrolytic breakdown of triglycerides to di and monoglycerides. Lower FA values guarantee a high-quality oil, showing it has been obtained from healthy olives and under ideal conditions [5,54]. PI measures the release of peroxide compounds arising from primary oxidation. The final stage in oxidation is the peroxide breakage, resulting in the formation of new compounds that contribute to oil rancidity. In turn, absorption coefficients K232 and K270 give information on the quantity of secondary oxidative compounds at 232 and 270 nm wavelengths. Lastly, ΔK is calculated as the difference between absorbance at 270 nm and 266–274 nm and is defined as a criterion to establish the purity and degradation degree of olive oil, which is correlated with the state of oxidation, and also detect possible adulterations with refined olive oil [54].

Results obtained concerning the olive oil quality parameters are given in Table 8. All the results of olive oil samples were compared to the ranges established for the highest quality category, “Extra Virgin Olive Oil”, in Regulation 1348/2013 [29]. These thresholds are 0.8% for acidity, 20 meq O_2_⋅kg^−1^ for PI, 2.50 for K232, 0.22 for K270 and 0.01 for ΔK. In the present study, almost all the values for the quality indices were below the established thresholds, except for PI obtained on FC treatment in 2018 and K232 obtained on both FC and BC olive oils in 2019.

Olive oil quality parameters were influenced by soil treatment and harvest year. In 2018, ZL was the treatment that showed the lowest values for FA, PI and K270 variables. However, BC olive oil showed the lowest value of ∆K. The higher level of PI obtained in FC olive oil indicates a higher level of peroxidation, which determines that this olive oil is not considered extra virgin. In 2019, olive oil provided from ZL tended to have the lowest quality parameters, namely FA, PI and K232. The values of K232 obtained for FC and BC were slightly higher than the defined threshold for EVOO characterization (2.50). Considering these results, olive oils obtained from FC showed the poorest quality. Among soil treatments, ZL stood out, showing olive oils with lower values of these quality indices, which in turn may indicate a lower level of oxidation and hydrolytic breakdown, and thus high VOO quality. There is some controversy in the literature concerning the relationship between these parameters and soil water availability. Some authors reported that these quality indices were more influenced by fruit and paste processing and manipulation than water availability [55]. However, it has also been described that higher FA, PI, K232 and K270 indices are common from drought and water deficit conditions [54,56]. This is in accordance with our results; at least once in 2019, the treatments with lower soil moisture (FC and BC) showed higher values of olive oil quality parameters when compared with ZL. Furthermore, it is important to highlight that the lower TP content observed in ZL olive oils did not compromise the olive oil quality. Probably, only the sensorial properties were influenced.

## 4. Conclusions

Olive fruit and oil polyphenolic composition were strongly modulated by the different soil treatments, confirming that different agronomic practices and soil properties influence the olive phenolic composition. The soil application of amendments, such as natural zeolites and biochar, changes soil physico-chemical and biological properties. In addition to ameliorating the soil quality and structure, it alters the plant secondary metabolism’s and polyphenolic composition. Therefore, adding soil conditioners together with chemical fertilizers appears to confer advantages to plant performance, evaluated by the tendency of the low accumulation of secondary metabolites while increasing the quality of olive fruit and VOO, compared with the isolated application of fertilizers. Among the applied sustainable soil amendments, in the short-term, natural zeolites appear to be an effective strategy to ameliorate fruit fatty acid composition and VOO quality and, at the same time, increase soil moisture. In turn, biochar was able to increase VOO nutritional value by the increase in polyphenols with benefits to human health. Despite the promising results of the present study, long-term studies are necessary to more accurately evaluate the effects on soil–plant interactions and olive fruit and VOO quality.

## Figures and Tables

**Table 1:** Climate characteristics recorded during 2018 and 2019 in the weather station at Paradela close to experimental plot. Average annual temperature (Tmean), maximum temperature (Tmax), minimum temperature (Tmin), average temperature from blossom to ripening period (May–October) (T mean (May–October)), cumulative annual precipitation (Σ Precp.) and cumulative precipitation from blossom to ripening period (May–October) (Σ Precp. (May–October)).

	T Mean (°C)	T Máx (°C)	T Min (°C)	T Mean _(May–October)_ (°C)	Ʃ Precep. (mm)	Ʃ Precep. _(May–October)_ (mm)
**2018**	13.2	39.1	−7.9	18.6	708.4	125.8
**2019**	13.0	34.3	−7.9	18.2	652.2	179.8

**Table 2:** Properties of the zeolites and biochar used in this study as provided by the manufacturers.

Zeolites		Biochar	
Particle size	0.6–1.5 mm	Particle size	0.1–10 mm
Bulk density	2000–2400 kg m^−3^	Bulk density	350–400 kg m^−3^
Cation-Exchange capacity	1.5–1.9 meq g^−1^	Moisture	≤30.0
Porosity	45.0–50.0%	Conductivity	948 µs cm^−1^
pH	7.0–8.0	pH	˂9.0
Specific surface	70.0–80.0 m^2^ g^−1^	Total organic C	≥90.0%
SiO_2_	65.0–72.0%	Ash	≤5.0%
Al_2_O_3_	10.0–12.0%	Volatile	≤5.0%
CaO	2.5–3.7%	Total N	≤5.0 g kg^−1^
K_2_O	2.3–3.5%	Cd	˂0.05 mg kg^−1^
Fe_2_O_3_	0.8–1.9%	Pb	0.05 mg kg^−1^
MgO	0.9–1.2%	Fe	99.5 mg kg^−1^
Na_2_O	0.3–0.65%	As	˂0.10 mg kg^−1^
TiO_2_	0.0–0.10%	Hg	˂0.10 mg kg^−1^

**Table 3:** Crop yield, fruit FW, pulp FW, pit FW, longitudinal length, equatorial length and Pulp/Pit ratio FW as a function of soil treatment and harvest year.

	Crop Yield (kg tree^−1^)	Fruit FW (g)	Pulp FW (g)	Pit FW (g)	Equat. Length (mm)	Long. Length (mm)	Pulp/Pit Ratio
**2018**							
FC	9.11 ± 1.54	3.53 ± 0.147 ^a^	2.69 ± 0.123	0.843 ± 0.026	16.1 ± 0.200 ^a^	22.5 ± 0.331 ^a^	3.24 ± 0.168
ZL	11.4 ± 1.19	3.08 ± 0.136 ^b^	2.28 ± 0.114	0.799 ± 0.025	15.3 ± 0.226 ^b^	21.3 ± 0.361 ^b^	2.83 ± 0.080
BC	10.8 ± 1.81	3.54 ± 0.150 ^a^	2.65 ± 0.129	0.885 ± 0.024	16.1 ± 0.217 ^a^	22.0 ± 0.376 ^ab^	2.98 ± 0.107
One-way ANOVA	n.s.	0.040	n.s.	n.s.	0.011	0.050	n.s.
**2019**							
FC	12.8 ± 1.55	4.27 ± 0.124	3.44 ± 0.107	0.829 ± 0.026	16.8 ± 0.188	24.3 ± 0.304	4.19 ± 0.109 ^ab^
ZL	10.9 ± 1.39	4.54 ± 0.143	3.71 ± 0.119	0.832 ± 0.027	17.5 ± 0.183	23.7 ± 0.263	4.47 ± 0.065 ^a^
BC	15.5 ± 2.81	4.19 ± 0.136	3.34 ± 0.117	0.830 ± 0.023	17.2 ± 0.198	23.6 ± 0.329	4.05 ± 0.085 ^b^
One-way ANOVA	n.s.	n.s.	n.s.	n.s.	n.s.	n.s.	0.004
Two-way ANOVA							
Year	n.s.	*p* < 0.0001	*p* < 0.0001	n.s.	*p* < 0.0001	0.017	*p* < 0.0001
Treatment	n.s.	n.s.	n.s.	n.s.	n.s.	*p* < 0.0001	n.s.
Year × Treatment	n.s.	0.007	0.004	n.s.	0.002	n.s.	0.0004

Values are means ± SEM. Significance by Tuckey HSD Test: *p* < 0.05. Means with different letters represent significant differences between treatments. n.s. represent non-significant differences between treatments.

**Table 4:** Total phenols (TP, mg GAE g^−1^ DW), ortho-diphenols (mg GAE g^−1^ DW), flavonoids (mg CE g^−1^ DW) and total antioxidant capacity (TAC, mmol TE g^−1^ DW) as function of soil management treatment and harvest year.

	Olive Fruits			Olive Oil		
	Ortho-Diphenols	Flavonoids	TAC	Ortho-Diphenols	Flavonoids	TAC
**2018**						
FC	41.9 ± 2.19 ^a^	23.1 ± 2.02	33.7 ± 1.11 ^a^	59.8 ± 2.22 ^b^	36.2 ± 7.06 ^b^	113.2 ± 2.87 ^b^
ZL	41.8 ± 1.79 ^a^	24.0 ± 1.73	30.9 ± 2.49 ^a^	54.7 ± 1.09 ^b^	35.4 ± 4.75 ^b^	123.6 ± 1.66 ^b^
BC	30.8 ± 1.74 ^b^	17.5 ± 1.93	14.7 ± 1.76 ^b^	110.1 ± 2.54 ^a^	150.3 ± 15.6 ^a^	186.2 ± 4.46 ^a^
One-way ANOVA	0.001	n.s.	*p* < 0.0001	*p* < 0.0001	*p* < 0.0001	*p* < 0.0001
**2019**						
FC	14.8 ± 0.933 ^b^	29.2 ± 3.27 ^b^	84.8 ± 3.04 ^b^	57.9 ± 0.918 ^ab^	77.7 ± 3.30 ^b^	249.4 ± 5.72 ^c^
ZL	12.8 ± 0.908 ^b^	32.8 ± 2.99 ^b^	98.9 ± 3.02 ^a^	62.2 ± 0.795 ^a^	109.7 ± 3.37 ^a^	307.7 ± 7.14 ^a^
BC	22.1 ± 1.37 ^a^	67.3 ± 5.77 ^a^	110.3 ± 5.11 ^a^	54.1 ± 2.68 ^b^	105.6 ± 1.75 ^a^	276.0 ± 7.75 ^b^
One-way ANOVA	*p* < 0.0001	*p* < 0.0001	0.001	0.024	*p* < 0.0001	*p* < 0.0001
Two-way ANOVA						
Year	*p* < 0.0001	0.0001	*p* < 0.0001	*p* < 0.0001	*p* < 0.0001	*p* < 0.0001
Treatment	n.s.	0.01	n.s.	*p* < 0.0001	*p* < 0.0001	*p* < 0.0001
Year × Treatment	*p* < 0.0001	0.0005	*p* < 0.0001	*p* < 0.0001	*p* < 0.0001	*p* < 0.0001

Values are means ± SEM. Significance by Tuckey HSD Test: *p* < 0.05. Means with different letters represent significant differences between treatments. n.s. represent non-significant differences between treatments.

**Table 5:** Olive fruit polyphenolic composition as function of soil management treatment and harvest year. Hydroxytyrosol (HyTyr); tyrosol (Tyr); chlorogenic acid (Chlorog.); verbascoside (Verb.); oleuropein (Oleur.); gallocatechin (GCT); epigallocatechin EGCT); epicatechin (EC); quercetin-3,7-di-*O*-glucoside (Querc.); luteolin-3,7-di-*O*-glucoside (Lut-3,7-di-*O*-gluc.); rutin; apigenin-7-*O*-glucoside (Api-7-*O*-gluc.); apigenin (Api.) and total phenolic compounds (TP) (mg kg^−1^ DW).

	Non-Flavonoid Composition					Flavonoid Composition								TP
	HyTyr	Tyr	Chlorog.	Verb.	Oleur.	GCT	EGCT	EC	Querc.	Lut-3,7-di-*O*-gluc.	Rutin	Api-7-*O*-gluc.	Api.	
**2018**														
FC	464.5 ± 7.62	9.39 ± 0.254 ^b^	256.7 ± 22.2 ^a^	175.7 ± 9.06 ^b^	2271.4 ± 84.4 ^b^	83.6 ± 6.38	243.8 ± 16.9 ^a^	134.9 ± 7.63 ^ab^	81.9 ± 2.19 ^a^	1630.1 ± 113.8	657.6 ± 77.2 ^b^	55.6 ± 6.19 ^b^	18.4 ± 1.15 ^b^	6319.4 ± 58.6 ^b^
ZL	459.0 ± 10.1	26.0 ± 0.698 ^a^	215.4 ± 6.14 ^a^	250.7 ± 25.7 ^a^	4274.0 ± 193.9 ^a^	88.5 ± 4.78	174.3 ± 5.30 ^b^	153.4 ± 5.07 ^a^	85.2 ± 0.239 ^a^	1541.1 ± 87.9	1100.2 ± 1.06 ^a^	83.9 ± 8.11 ^a^	21.9 ± 0.977 ^b^	8648.9 ± 271.0 ^a^
BC	340.3 ± 81.2	28.8 ± 2.88 **^a^**	93.0 ± 3.66 ^b^	167.2 ± 1.59 ^b^	1471.9 ± 170.7 ^c^	82.5 ± 10.9	78.7 ± 7.54 ^c^	129.5 ± 0.911 ^b^	52.9 ± 2.06 ^b^	1527.7 ± 69.8	785.2 ± 42.4 ^b^	95.9 ± 3.02 ^a^	42.8 ± 0.922 ^a^	5003.1 ± 200.2 ^c^
One-way ANOVA	n.s.	*p* < 0.0001	*p* < 0.0001	0.018	*p* < 0.0001	n.s.	*p* < 0.0001	0.043	*p* < 0.0001	n.s.	0.002	0.009	*p* < 0.0001	*p* < 0.0001
**2019**														
FC	494.9 ± 7.16 ^a^	13.9 ± 0.141 ^a^	127.1 ± 3.53 ^ab^	407.9 ± 5.30 ^a^	1688.9 ± 44.2 ^a^	115.2 ± 6.11 ^a^	58.5 ± 3.32 ^c^	131.8 ± 1.68 ^a^	26.9 ± 0.170 ^b^	652.1 ± 8.80 ^b^	364.2 ± 12.5 ^a^	157.0 ± 2.72 ^b^	18.8 ± 0.437	4657.0 ± 39.0 ^a^
ZL	328.5 ± 16.2 ^b^	8.89 ± 0.403 ^b^	146.0 ± 5.73 ^a^	105.4 ± 5.17 ^b^	1031.2 ± 27.9 ^b^	106.1 ± 1.64 ^ab^	106.2 ± 2.15 ^b^	109.5 ± 3.11 ^b^	33.5 ± 0.789 ^a^	1162.2 ± 42.5 ^a^	381.9 ± 11.9 ^a^	126.1 ± 4.47 ^c^	15.2 ± 2.21	3832.2 ± 107.2 ^b^
BC	296.3 ± 7.26 ^b^	13.8 ± 0.635 ^a^	106.7 ± 7.34 ^b^	95.6 ± 4.85 ^b^	996.8 ± 49.1 ^b^	94.6 ± 1.69 ^b^	179.7 ± 4.19 ^a^	129.2 ± 4.72 ^a^	33.1 ± 0.237 ^a^	1074.1 ± 100.1 ^a^	317.4 ± 5.63 ^b^	392.7 ± 0.114 ^a^	16.0 ± 1.10	3679.1 ± 85.8 ^b^
One-way ANOVA	*p* < 0.0001	*p* < 0.0001	0.009	*p* < 0.0001	*p* < 0.0001	0.024	*p* < 0.0001	0.007	*p* < 0.0001	0.003	0.012	*p* < 0.0001	n.s.	*p* < 0.0001
Two-way ANOVA														
Year	n.s.	*p* < 0.001	*p* < 0.001	n.s.	*p* < 0.001	0.002	*p* < 0.001	0.001	*p* < 0.001	*p* < 0.001	*p* < 0.001	*p* < 0.001	*p* < 0.001	*p* < 0.001
Treatment	0.002	*p* < 0.001	*p* < 0.001	*p* < 0.001	*p* < 0.001	n.s.	n.s.	n.s.	*p* < 0.001	n.s.	*p* < 0.001	*p* < 0.001	*p* < 0.001	*p* < 0.001
Year × Treatment	n.s.	*p* < 0.001	*p* < 0.001	*p* < 0.001	*p* < 0.001	n.s.	*p* < 0.001	0.0006	*p* < 0.001	0.005	0.0003	*p* < 0.001	*p* < 0.001	*p* < 0.001

Values are means ± SEM. Significance by Tukey HSD Test: *p* < 0.05. Means with different letters represent significant differences between treatments. n.s. represent non-significant differences between treatments.

**Table 6:** Olive oil polyphenolic composition as function of soil management treatment and harvest year. 3,4-Dihydroxyphenylglycol (DHPG); hydroxytyrosol (HyTyr); tyrosol (Tyr); hydroxytyrosol acetate (HyTyr acet.); oleuropein aglycone (Oleur. aglyc.); 3,4-DHPEA-EDA (dialdehydic forms of decarboxymethyl elenolic acid linked to hydroxytyrosol); verbascoside (Verb.); oleuropein (Oleur.); luteolin-7-*O*-glucoside (Lut.-7-*O*-gluc.); luteolin-3,7-di-*O*-glucoside (Lut.-3,7-di-*O*-gluc.); rutin; apigenin-7-*O*-glucoside (Api.-7-*O*-gluc.); luteolin (Lut.); apigenin (Api.) and total phenols (TP) (mg kg^−1^ DW).

	Non-Flavonoid Composition								Flavonoid Composition						TP
	DHPG	HyTyr	Tyr	HyTyr Acet.	Oleur. Aglyc.	3,4-DHPEA-EDA	Verb.	Oleur.	Lut.7-O-gluc.	Lut.-3,7-di-*O*-gluc.	Rutin	Api-7-*O*-gluc.	Lut.	Api.	
**2018**															
FC	0.161 ± 0.004 ^b^	0.048 ± 0.0002 ^b^	0.194 ± 0.0006 ^b^	0.059 ± 0.001	13.5 ± 0.202	0.558 ± 0.001 ^c^	0.271 ± 0.001 ^b^	0.530 ± 0.276 ^b^	0.030 ± 0.002 ^c^	0.175 ± 0.0002 ^c^	1.65 ± 0.010 ^c^	0.211 ± 0.005 ^b^	11.4 ± 0.128 ^b^	6.39 ± 0.151 ^c^	52.5 ± 0.384 ^c^
ZL	0.089 ± 0.003 ^c^	0.052 ± 0.0004 ^a^	0.075 ± 0.006 ^c^	0.077 ± 0.001	13.1 ± 0.095	0.957 ± 0.009 ^a^	0.299 ± 0.007 ^c^	1.17 ± 0.022 ^a^	0.311 ± 0.001 ^a^	0.234 ± 0.004 ^b^	1.72 ± 0.019 ^b^	0.149 ± 0.004 ^c^	11.3 ± 0.749 ^b^	7.95 ± 0.165 ^a^	61.1 ± 0.325 ^b^
BC	0.205 ± 0.002 ^a^	0.045 ± 0.001 ^c^	0.530 ± 0.035 ^a^	0.074 ± 0.003	13.6 ± 0.472	0.700 ± 0.001 ^b^	0.402 ± 0.005 ^a^	1.26 ± 0.025 ^a^	0.070 ± 0.001 ^b^	0.264 ± 0.009 ^a^	1.86 ± 0.014 ^a^	0.915 ± 0.003 ^a^	17.5 ± 0.499 ^a^	7.35 ± 0.44 ^b^	64.5 ± 0.471 ^a^
One-way ANOVA	*p* < 0.0001	0.002	*p* < 0.0001	n.s.	n.s.	*p* < 0.0001	*p* < 0.0001	*p* < 0.0001	*p* < 0.0001	*p* < 0.0001	*p* < 0.0001	*p* < 0.0001	*p* < 0.0001	*p* < 0.0001	*p* < 0.0001
**2019**															
FC	0.171 ± 0.007 ^b^	0.012 ± 0.0003	1.01 ± 0.026	0.033 ± 0.002 ^b^	8.68 ± 0.523 ^b^	0.045 ± 0.002 ^b^	0.176 ± 0.005	0.872 ± 0.014 ^b^	0.009 ± 0.0001	0.103 ± 0.002 ^c^	1.33 ± 0.010 ^b^	2.42 ± 0.052 ^a^	27.2 ± 1.16 ^a^	9.80 ± 0.425 ^a^	60.4 ± 1.55 ^a^
ZL	0.147 ± 0.001 ^b^	0.011 ± 0.001	0.849 ± 0.016	0.034 ± 0.002 ^b^	7.57 ± 0.054 ^b^	0.041 ± 0.002 ^b^	0.173 ± 0.006	1.09 ± 0.049 ^ab^	0.016 ± 0.004	0.173 ± 0.001 ^a^	1.35 ± 0.004 ^b^	1.86 ± 0.014 ^b^	16.1 ± 0.785 ^b^	6.29 ± 0.129 ^b^	44.2 ± 0.458 ^c^
BC	0.212 ± 0.016 ^a^	0.013 ± 0.0003	0.969 ± 0.069	0.047 ± 0.002 ^a^	11.4 ± 0.416 ^a^	0.077 ± 0.006 ^a^	0.198 ± 0.009	1.15 ± 0.079 ^a^	0.008 ± 0.0001	0.142 ± 0.008 ^b^	1.47 ± 0.013 ^a^	1.79 ± 0.125 ^b^	17.8 ± 0.045 ^b^	6.38 ± 0.183 ^b^	53.8 ± 0.602 ^b^
One-way ANOVA	0.012	n.s.	n.s.	0.001	0.001	0.001	n.s.	0.026	n.s.	*p* < 0.0001	*p* < 0.0001	0.003	*p* < 0.0001	*p* < 0.0001	*p* < 0.0001
Two-way ANOVA															
Year	0.002	*p* < 0.001	*p* < 0.001	*p* < 0.001	*p* < 0.001	*p* < 0.001	*p* < 0.001	n.s.	*p* < 0.001	*p* < 0.001	*p* < 0.001	*p* < 0.001	*p* < 0.001	n.s.	*p* < 0.001
Treatment	*p* < 0.0001	0.0027	*p* < 0.001	0.0035	0.0001	*p* < 0.001	*p* < 0.001	*p* < 0.0001	*p* < 0.001	*p* < 0.001	*p* < 0.001	*p* < 0.001	*p* < 0.001	0.0002	*p* < 0.001
Year × Treatment	0.009	*p* < 0.001	0.0002	0.047	0.001	*p* < 0.001	*p* < 0.001	0.0003	*p* < 0.001	*p* < 0.001	n.s.	*p* < 0.001	*p* < 0.001	*p* < 0.0001	*p* < 0.001

Values are means ± SEM. Significance by Tuckey HSD Test: *p* < 0.05. Means with different letters represent significant differences between treatments. n.s. represent non-significant differences between treatments.

**Table 7:** Olive fruit fat content (%) and fatty acid profile (%) as function of soil management treatment and harvest year. Fat content, palmitic acid (C16:0), palmitoleic acid (C16:1), oleic acid (C16:1), linoleic acid (C18:2), linolenic acid (C18:3), saturated fatty acids (SFA), unsaturated fatty acids (UFA), monounsaturated fatty acids (MUFA), polyunsaturated fatty acids (PUFA), UFA/SFA and oleic/linoleic acid ratio.

	Fat Content	Palmitic Acid	Palmitoleic Acid	Oleic Acid	Linoleic Acid	Linolenic Acid	SFA	MUFA	PUFA	Oleic/Linoleic
**2018**										
FC	56.4 ± 0.742	14.4 ± 0.395	0.909 ± 0.049	72.3 ± 0.162 ^b^	9.24 ± 0.189 ^a^	0.649 ± 0.008	16.7 ± 0.305	73.4 ± 0.115 ^b^	9.89 ± 0.181 ^a^	7.84 ± 0.143 ^b^
ZL	56.9 ± 0.376	14.1 ± 0.020	0.992 ± 0.002	73.4 ± 0.114 ^a^	8.32 ± 0.112 ^b^	0.666 ± 0.003	16.5 ± 0.013	74.5 ± 0.123 ^a^	8.99 ± 0.109 ^b^	8.81 ± 0.132 ^a^
BC	60.6 ± 1.98	14.4 ± 0.109	1.03 ± 0.039	72.0 ± 0.141 ^b^	9.41 ± 0.083 ^a^	0.647 ± 0.013	16.9 ± 0.127	73.2 ± 0.109 ^b^	10.1 ± 0.090 ^a^	7.66 ± 0.079 ^b^
One-way ANOVA	n.s.	n.s.	n.s.	0.001	0.003	n.s.	n.s.	*p* < 0.0001	0.003	0.001
**2019**										
FC	60.3 ± 1.01 ^a^	13.3 ± 0.034 ^a^	0.781 ± 0.022	75.9 ± 0.483 ^a^	7.67 ± 0.157	0.549 ± 0.001 ^b^	15.7 ± 0.086 ^a^	76.8 ± 0.471 ^b^	8.22 ± 0.158	9.91 ± 0.140
ZL	56.1 ± 0.440 ^b^	13.3 ± 0.081 ^a^	0.841 ± 0.040	73.8 ± 0.255 ^b^	8.56 ± 0.326	0.684 ± 0.017 ^a^	15.9 ± 0.044 ^a^	74.9 ± 0.299 ^c^	9.24 ± 0.342	8.65 ± 0.359
BC	62.4 ± 0.718 ^a^	12.5 ± 0.197 ^b^	0.724 ± 0.032	76.7 ± 0.147 ^a^	7.88 ± 0.386	0.711 ± 0.021 ^a^	14.9 ± 0.152 ^b^	77.6 ± 0.105 ^a^	8.59 ± 0.364	9.79 ± 0.469
One-way ANOVA	0.003	0.007	n.s.	0.002	n.s.	0.001	0.001	0.003	n.s.	n.s.
Two-way ANOVA										
Year	n.s.	*p* < 0.0001	*p* < 0.0001	*p* < 0.0001	0.0003	n.s.	*p* < 0.0001	*p* < 0.0001	0.0003	*p* < 0.0001
Treatment	0.0013	n.s.	n.s.	0.027	n.s.	*p* < 0.0001	n.s.	0.038	n.s.	n.s.
Year × Treatment	n.s.	0.038	0.042	*p* < 0.0001	0.003	0.0001	0.0009	*p* < 0.0001	0.003	0.001

Values are means ± SEM. Significance by Tuckey HSD Test: *p* < 0.05. Means with different letters represent significant differences between treatments. n.s. represent non-significant differences between treatments.

**Table 8:** Olive oil quality indices as function of soil management treatments and harvest year. Free Acidity (FA, %), peroxide index (PI, mEq of O_2_ kg^−1^), K232, K270 and ΔK.

	FA	PI	K232	K270	∆K
**2018**					
FC	0.221 ± 0.0002 ^b^	20.2 ± 0.735 ^a^	2.18 ± 0.092 ^a^	0.119 ± 0.002 ^ab^	0.004 ± 0.0002 ^ab^
ZL	0.201 ± 0.0002 ^c^	9.79 ± 0.864 ^b^	2.06 ± 0.016 ^a^	0.113 ± 0.003 ^b^	0.003 ± 0.00002 ^b^
BC	0.279 ± 0.001 ^a^	10.4 ± 0.568 ^b^	1.68 ± 0.051 ^b^	0.133 ± 0.006 ^a^	0.0004 ± 0.0001 ^a^
One-way ANOVA	*p* < 0.0001	*p* < 0.0001	0.003	0.040	0.030
**2019**					
FC	0.161 ± 0.0001 ^a^	2.54 ± 0.209 ^a^	2.92 ± 0.006 ^a^	0.185 ± 0.001	0.004 ± 0.0002
ZL	0.121 ± 0.0001 ^b^	1.69 ± 0.004 ^b^	2.46 ± 0.054 ^b^	0.181 ± 0.001	0.038 ± 0.00001
BC	0.121 ± 0.0001 ^b^	2.50 ± 0.052 ^a^	2.67 ± 0.125 ^ab^	0.188 ± 0.002	0.004 ± 0.0001
One-way ANOVA	*p* < 0.0001	0.005	0.017	0.053	n.s.
Two-way ANOVA					
Year	*p* < 0.0001	*p* < 0.0001	*p* < 0.0001	*p* < 0.0001	0.0015
Treatment	*p* < 0.0001	*p* < 0.0001	0.0005	0.0040	0.0049
Year × Treatment	*p* < 0.0001	*p* < 0.0001	0.005	n.s.	n.s.

Values are means ± SEM. Significance by Tuckey HSD Test: *p* < 0.05. Means with different letters represent significant differences between treatments. n.s. represent non-significant differences between treatments.

## Data Availability

The data presented in this study are available in the article and Supplementary Materials.

## Supplementary Materials

The following supporting information can be downloaded at: https://www.mdpi.com/article/10.3390/antiox11071332/s1, Table S1: CV-ANOVA respective to the OPLS-DA of olive fruit phenolic compounds; Figure S1: Permutation tests of OPLS-DA relative to the olive fruit phenolic compounds according to FC (a), ZL (b) and BC (c) treatments; Table S2: CV-ANOVA respective to the OPLS-DA of olive oil phenolic compounds; Figure S2: Permutation tests of OPLS-DA relative to the olive oil phenolic compounds according to FC (a), ZL (b) and BC (c) treatments; Figure S3: Correlation between the olive fruit total phenolic compounds in relation to olive oil phenolic compounds; Table S3: CV-ANOVA respective to the OPLS-DA of olive fruit fatty acids; Figure S4: Permutation tests of OPLS-DA relative to the olive fruit fatty acids, according to FC (a), ZL (b) and BC (c) treatments.
