# Supplementary material for: Zeolites and Biochar Modulate Olive Fruit and Oil Polyphenolic Profile
Source: Antioxidants (Basel). 2022 Jul 6;11(7):1332. doi: 10.3390/antiox11071332 (PMC9311664; doi:10.3390/antiox11071332)
Supplement: Supplementary file 1 [file antioxidants-11-01332-s001.zip › antioxidants-1768249-supplementary.pdf]

Supplementary Results

Table S1. CV-ANOVA respective to the OPLS-DA of olive fruit phenolic compounds.

M1(Untitled)	SS	DF	MS	F	*p*	SD
Total Corr.	34	34	1			1
Regression	25.8987	18	1.43882	2.84167	0.020472	1.19951
Residual	8.10126	16	0.506329			0.711568

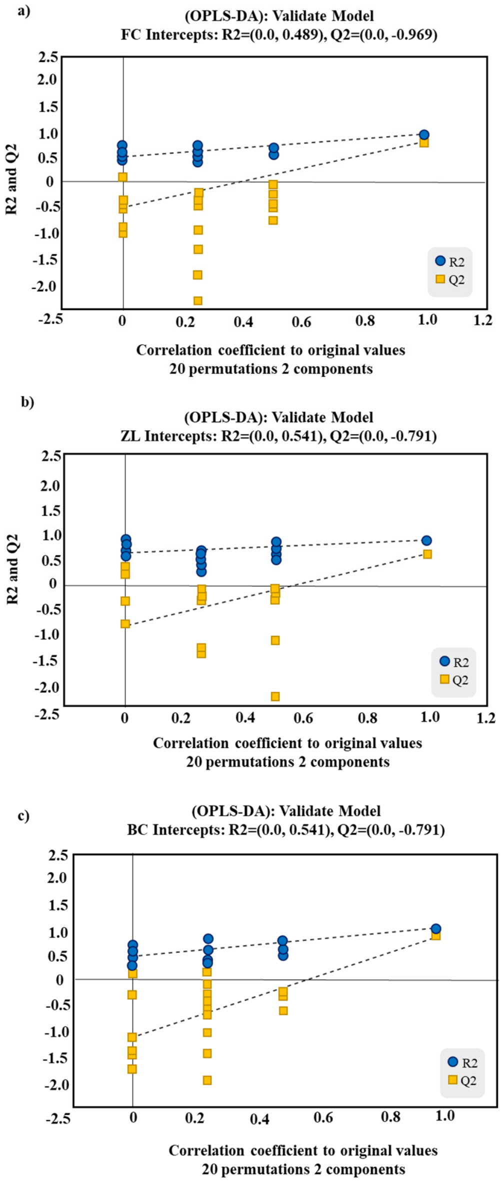

Figure S1. Permutation tests of OPLS-DA relative to the olive fruit phenolic compounds according to FC **(a)**, ZL **(b)** and BC **(c)** treatments

Table S2. CV-ANOVA respective to the OPLS-DA of olive oil phenolic compounds.

M1(Untitled)	SS	DF	MS	F	*p*	SD
Total Corr.	34	34	1			1
Regression	30.2492	14	2.16066	11.5212	1.13564×10^{-4}	1.46992
Residual	3.75076	20	0.187538			0.43057

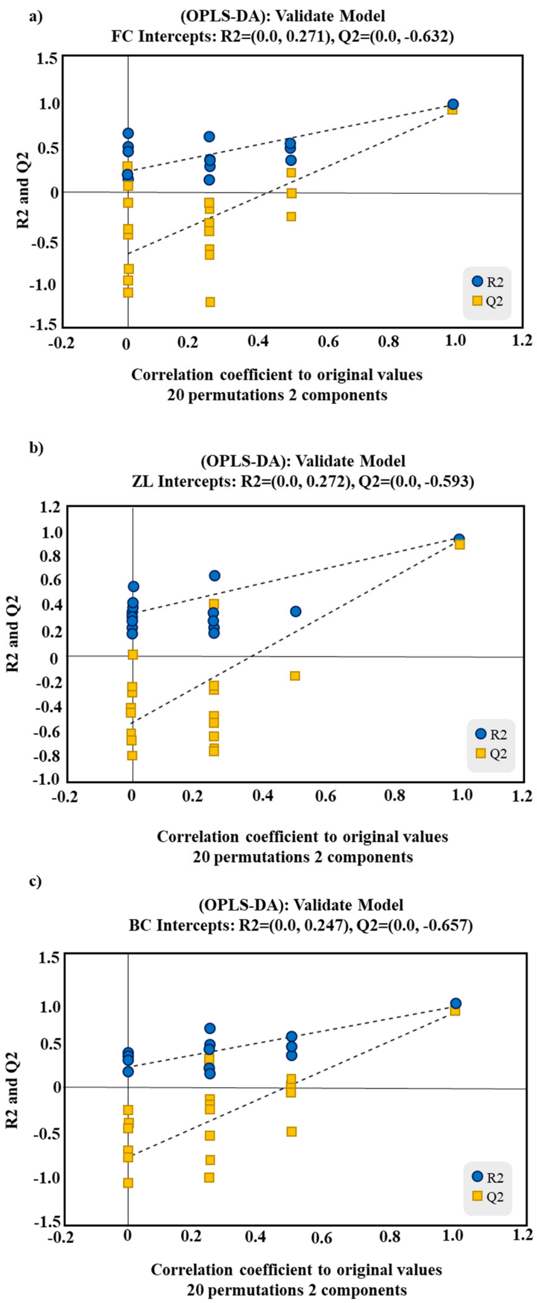

Figure S2. Permutation tests of OPLS-DA relative to the olive oil phenolic compounds according to FC **(a)**, ZL **(b)** and BC **(c)** treatments.

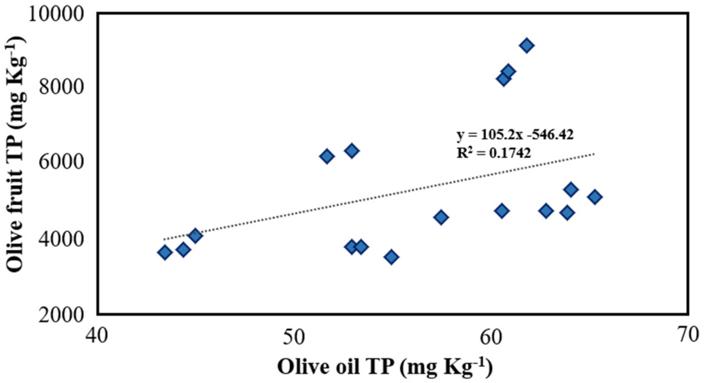

Figure S3. Correlation between the olive fruit total phenolic compounds in relation to olive oil phenolic compounds.

Table S3. CV-ANOVA respective to the OPLS-DA of olive fruit fatty acids.

M1(Untitled)	SS	DF	MS	F	*p*	SD
Total Corr.	34	34	1			1
Regression	0	12	0	0	1	
Residual	36.6921	22	1.66782			1.29144

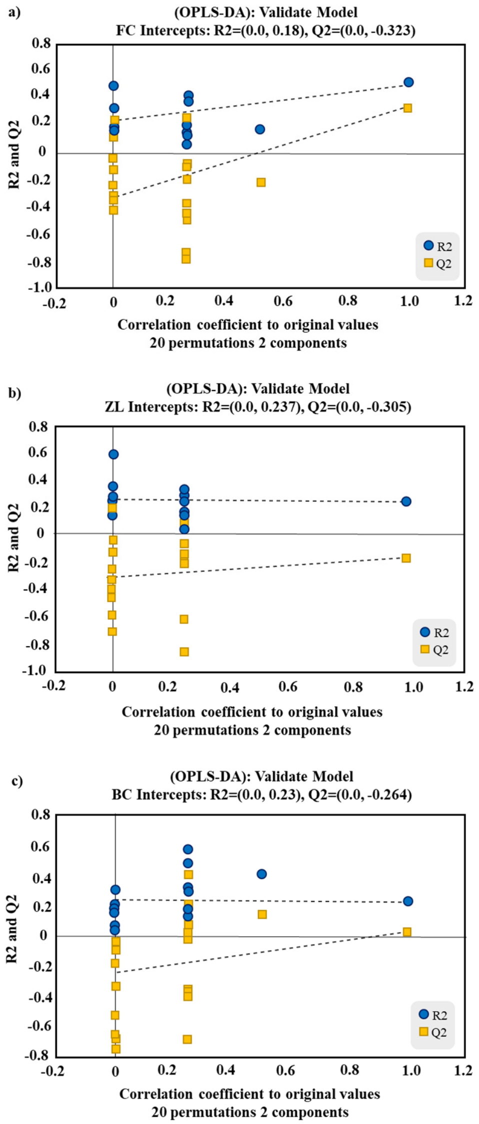

Figure S4. Permutation tests of OPLS-DA relative to the olive fruit fatty acids, according to FC **(a)**, ZL **(b)** and BC **(c)** treatments.
